# Genome-wide identification of *GATA* family genes in sweet potato (*Ipomoea batatas* L.) and their expression patterns under abiotic stress

**DOI:** 10.3389/fgene.2025.1635749

**Published:** 2025-07-09

**Authors:** Chong Wang, Mengjiao Lan, Manqiu Xiao, Ying Peng, Hao Pan, Jiaqi Deng, Wensheng Wu

**Affiliations:** ^1^ Jiangxi Province Key Laboratory of Oil Crops Genetic Improvement, Institute of Crops, Jiangxi Academy of Agricultural Sciences, Nanchang, China; ^2^ Institute of Food Crops, Hubei Academy of Agricultural Sciences, Wuhan, China; ^3^ College of Agriculture, Yangtze University, Jingzhou, China

**Keywords:** sweet potato (*Ipomoea batatas* L.), GATA transcription factor, genome-wide, abiotic stress, drought and salt stress

## Abstract

GATAs, a type of zinc finger protein transcription factors, can bind to DNA regulatory regions to control the expression of target genes, thereby affecting plant growth and development under normal conditions or environmental stress. However, the *GATA* gene family has not been identified in sweet potato. In this study, a total of 35, 33, 34, 39, 63, and 56 *GATA* genes were identified in sweet potato, *Ipomoea aquatica*, *Ipomoea cairica*, *Ipomoea nil*, *Ipomoea triloba*, and *Ipomoea trifida*, respectively. Phylogenetic analysis categorized the *GATA* genes into six groups according to their distinct features, and this classification was validated by the structural characteristics of exons/introns and conserved motif analysis. The *cis*-acting elements located in the promoter regions were also found to be enriched with biotic and abiotic responsive elements, which may play a pivotal role in plant stress adaptation. Then the gene duplication events and synteny between the genome of sweet potato and those of *Ipomoea aquatica*, *Ipomoea cairica*, *Ipomoea nil*, *Ipomoea triloba*, and *Ipomoea trifida* were analyzed, which provided insights into evolutionary mechanisms. Moreover, expression pattern analysis was performed on *IbGATA* genes, many of which were significantly induced by multiple types of abiotic stress, which may render these genes candidates for molecular breeding strategies in sweet potato. Overall, this experiment conducted a systematic exploration of *GATA* genes by investigating their evolutionary relationships, structural characteristics, functional properties, and expression patterns, thereby establishing a theoretical foundation for further in-depth research on the features of the *GATA* gene family.

## 1 Introduction

Transcription factors (TFs) are a class of protein factors that regulate gene expression by binding to specific *cis*-acting regulatory elements in the promoter regions of downstream target genes, which play important roles in plant development and stress response (Strader et al., 2022). There are abundant and diverse TFs distributed in plants. The study of TFs is important for understanding the genetic regulation of gene expression in multiple metabolic pathways in plants. A growing family of TFs have been identified in plants, such as MYB (myeloblastosis) (Dubos et al., 2010; Chen et al., 2019; Millard et al., 2019; Zhang et al., 2023), bZIP (basic leucine zipper) (Liu et al., 2023), AP2/ERF (APETALA2/ethylene responsive factor) (Feng et al., 2020; Ma et al., 2024), bHLH (basic helix–loop–helix) (Gao and Dubos, 2024; Lei et al., 2024), NAC (NAM, ATAF1/2, CUC) (Yan et al., 2021; Yan et al., 2023), WRKY (Chen et al., 2018; Mahiwal et al., 2024), and GATA (Du et al., 2022).

GATA TFs are transcription regulatory factors found in animals, plants, and fungi that recognize the DNA sequence W-G-A-T-A-R through a single type IV zinc finger and regulate the transcriptional levels of the target genes (Patient and McGhee, 2002). GATA TFs were first discovered and reported to bind globin gene promoters in chickens and participate in the hematopoietic process. Subsequent studies have shown that GATA TFs in animals contain two C-X_2_-C-X_17_-_20_-C-X_2_-C zinc finger domains, in which only the C-terminal zinc finger structure binds to DNA, whereas the N-terminal zinc finger structure regulates the specific binding of the C-terminal zinc finger to DNA and participates in the process of development, differentiation, and cell proliferation. Most of the GATA TFs in fungi contain only one zinc finger domain, divided into two classes, namely C-X_2_-C-X_17_-C-X_2_-C or C-X_2_-C-X_18_-C-X_2_-C domains, which play a key role in a variety of biological processes, such as light induction, circadian rhythm, siderophore biosynthesis, main-type switching, and nitrogen cycling. In plants, the first known GATA TF was identified in tobacco (*Nicotiana tabacum*) and named NTL1 because it is a homolog of the NIT2 protein found in *Neurospora crassa* (Daniel-Vedele and Caboche, 1993). GATA TFs have since been studied in numerous plants, such as rice (*Oryza sativa*) (Reyes et al., 2004), tomato (*Lycopersicon esculentum*) (Zhao et al., 2021), soybean (*Glycine max*) (Zhang et al., 2020), and potato (*Solanum tuberosum*) (Aksoy et al., 2024).

Most plant GATA proteins have a single C-X_2_-C-X_18_-C-X_2_-C zinc finger structural domain, whereas only a few GATA proteins have C-X_2_-C-X_20_-C-X_2_-C or two zinc finger structural domains. It has been shown that GATA TFs play important roles in regulating plant growth and development and nitrogen metabolism, as well as in mediating responses to biotic and abiotic stresses. For instance, the *Arabidopsis* GATA TF BME3 mediates the developmental processes of seeds from dormancy to germination and positively regulates seed germination (Liu et al., 2005). In *Arabidopsis thaliana*, GATA TF ZIM regulates hypocotyl and petiole elongation, whereas overexpression of GATA TF TaZIM-A1 in *Triticum aestivum* leads to delayed flowering and decreased thousand-grain weight (Shikata et al., 2004; Liu et al., 2019). GATA TFs have been found to play important roles in plant photomorphogenesis, of which AtGATA2 is an important positive regulator of photomorphogenesis, which can directly bind to the promoter of photoresponsive genes and brassinosteroid (BR) genes to regulate their expression (Luo et al., 2010). In *Arabidopsis thaliana,* GATA TFs GNC (GATA, nitrate-inducible, carbon metabolism-involved) and GNL (GNC-like) regulate chlorophyll synthesis, flowering time, and cold resistance (Richter et al., 2013). GNC and GNL help balance the phototropic and gravitropic growth responses in *Arabidopsis thaliana* (Sala et al., 2023). In rice, overexpression of *OsGATA6* resulted in delayed heading, increased grain number, and decreased grain size, which potentially increases rice yield (Zhang et al., 2022). *OsGATA8* increases seed size and stress resistance in both *Arabidopsis* and rice by regulating the expression of critical genes involved in stress tolerance, scavenging of reactive oxygen species, and chlorophyll biosynthesis (Nutan et al., 2020). Moreover *OsGATA8* has also been found to be a key coordinator of uptake and tiller formation in rice. *OsGATA8* negatively regulates nitrogen uptake by repressing the transcription of the ammonium transport gene *OsAMT3.2*. At the same time, it promotes the formation of tillers by inhibiting the transcription of *OsTCP19* (Wu et al., 2024). The OsGATA16 positive regulator controls chlorophyll biosynthesis and chloroplast development by directly binding to the promoter regions of *OsHEMA*, *OsCHLH*, *OsPORA*, *OsPORB*, and *OsFtsZ* and upregulates their expression. Meanwhile, it improves cold tolerance at the seedling stage in rice by binding to the promoter region of *OsWRKY45-1* and repressing its expression (Lim et al., 2024; Zhang et al., 2021). The functions of GATA TFs have also been discovered and identified in other plants. In potato, *StGATA2* enhances the ability of potato to resist heat damage (Zhu et al., 2023). In tomato, *SlGATA17* promotes drought tolerance of transgenic tomato by enhancing the activity of the phenylpropanoid biosynthesis pathway (Zhao et al., 2021). *PdGNC* and *PdGATA19* regulate photosynthesis, growth, and drought resistance in poplars (Shen et al., 2021; An et al., 2020).

Sweet potato (*Ipomoea batatas* L.), the seventh most valuable crop in the world, is a fundamental source of calories, protein, vitamins, and minerals for humans (Yang et al., 2017). Sweet potato is widely cultivated in various countries and regions around the world and plays a vital role in food security, hunger eradication, nutrition provision, and poverty reduction in poverty-stricken areas for its adaptability and resilience to different planting environments and soil conditions (Wu et al., 2018). Sweet potato is usually cultivated in marginal areas such as desert margins, coastal mudflats, and hilly area, and drought and salt stress are limiting factors inhibiting its growth and yield. The sweet potato stress tolerance-related TFs *IbMYB308* (Wang et al., 2022), *IbC3H18* (Zhang et al., 2019), *IbBBX24* (Zhang et al., 2022), and *IbNAC3* (Meng et al., 2023) have been reported successively. With the rapid development of sequencing technology, more and more families of TFs have been identified in plants. Currently, *GATA* gene families have been identified in many plants, including *Arabidopsis thaliana* (Kim et al., 2021), *Triticum aestivum* (Feng et al., 2022), *Capsicum annuum* (Yu et al., 2021), *Solanum tuberosum* (Zhang et al., 2024), and *Setaria italica* (Lai et al., 2022). Based on the whole-genome-wide analyses, 33, 64, and 96 *GATA* family genes were identified in *Sorghum bicolor*, *Glycine max*, and *Brassica napus*, respectively (Yao et al., 2023; Zhang et al., 2015; Zhu et al., 2020). However, the identification, classification, evolution, and function of the *GATA* gene family remain unclear in sweet potato.

In this study, the *GATA* gene family in the whole genome of sweet potato was identified using bioinformatic methods. Then the physicochemical properties, chromosomal distributions, gene structure, conserved motifs, duplication events, phylogenetic relationships, and expression profiles of *GATA* genes in different tissues and multiple adversity stresses were analyzed. It provides a theoretical basis for further studying the functions of *GATA* gene family members and provides references for molecular breeding of sweet potato.

## 2 Materials and methods

### 2.1 Identification of *GATA* genes in *Ipomoea* species

The whole-genome sequence and annotation files of *Ipomoea batatas*, *Ipomoea trifida*, and *Ipomoea triloba* were downloaded from the Ipomoea Genome Hub (https://sweetpotato.com/) and Sweetpotato Genomics Resource (http://sweetpotato.uga.edu/), respectively. The whole genome information of *Ipomoea cairica*, *Ipomoea aquatica*, and *Ipomoea nil* was downloaded from Plant GARDEN (https://plantgarden.jp/). The genome annotations of *Arabidopsis thaliana* were downloaded from TAIR (https://www.arabidopsis.org/). To identify the sweet potato *GATA* genes, the *Arabidopsis GATA* gene family was obtained from PlantTFDB 5.0 (https://planttfdb.gao-lab.org/). The IbGATA proteins were identified using two screening methods. First, based on the amino acid sequence of 30 AtGATA members in *Arabidopsis*, BLAST (E-value ≤ 1e-5) searches were performed in sweet potato protein sequences to identify candidate IbGATA proteins. Then the hidden Markov model file of the GATA protein domain (PF00320) was downloaded from the Pfam dataset (http://pfam.xfam.org/), and the whole sweet-potato protein sequence was retrieved using HMMER 3.3.2 software, and the screening threshold was set at an E-value ≤ 1e-5. Finally, duplicate redundant sequences were eliminated by combining the search results of both methods. To ensure the reliability of the candidate sequences, the integrity of their conserved domains was verified using SMART (http://smart.embl-heidelberg.de/) and the NCBI-CDD search program (https://www.ncbi.nlm.nih.gov/Structure/cdd/wrpsb.cgi). Proteins that were absent in the GATA structural domain were manually eliminated to obtain the final IbGATA proteins. The important physicochemical properties of the identified proteins, such as protein sequence length, molecular weight (MW), and theoretical isoelectric point (pI), were analyzed using the online ExPASy program (https://www.expasy.org/). The subcellular localization predictions of IbGATA proteins were predicted on the online website Cell-PLoc 2.0 (http://www.csbio.sjtu.edu.cn/bioinf/Cell-PLoc-2/).

### 2.2 Gene structure, protein motifs, and conserved domain analysis of *GATA* genes

Gene structure information of *IbGATAs* was extracted from sweet potato gff3 files, and a visual map of the gene structure was mapped using TBtools software (Chen et al., 2023). Conserved motifs in IbGATA proteins were discovered using the online tool MEME (https://meme-suite.org/meme/tools/meme); the maximum number of motifs was set to 10, and the remaining parameters were set to default values. The conserved domain of IbGATA proteins was verified using the NCBI-CDD database (https://www.ncbi.nlm.nih.gov/cdd/).

### 2.3 *Cis*-acting elements in the promoter region of *IbGATA* genes

The promoter sequences 2,000 bp upstream of the start codon of *IbGATA* genes were extracted from sweet potato genome data using TBtools software (Chen et al., 2023). Then *cis*-acting elements on the promoter sequences were predicted and screened using the online website PlantCARE (https://bioinformatics.psb.ugent.be/webtools/plantcare/html/) and visualized on TBtools software (Chen et al., 2023).

### 2.4 Phylogenetic analysis of *GATA* genes

Multiple sequence alignment of GATA proteins from *Arabidopsis thaliana*, *Oryza sativa*, *Ipomoea cairica*, *Ipomoea aquatica*, *Ipomoea trifida*, *Ipomoea triloba*, and *Ipomoea nil* with identified GATA proteins from sweet potato was performed using ClustalW in MEGA X (Kumar et al., 2018). The obtained aligned sequences were submitted to MEGA X software (Kumar et al., 2018) for phylogenetic analysis, and the phylogenetic tree self-expansion value was set to 1,000, with the rest set to default. Afterward, the obtained phylogenetic tree was embellished and modified in Evolview (Subramanian et al., 2019).

### 2.5 Chromosomal localization and collinearity analysis of *GATA* genes

The information about *GATA* gene positions in chromosomes was obtained from gff3 files of *Ipomoea* species and then mapped on the chromosomes using TBtools software (Chen et al., 2023). The syntenic relationship of orthologous *GATA* genes between *Ipomoea batatas* and other *Ipomoea* species, *Ipomoea aquatica*, *Ipomoea cairica*, *Ipomoea nil*, *Ipomoea triloba*, and *Ipomoea trifida*, was analyzed using MCScanX software (Chen et al., 2023).

### 2.6 Ka/Ks analysis of duplicate and synonymous *GATA* genes

The non-synonymous substitution rate (Ka), synonymous substitution rate (Ks), and the ratio (Ka/Ks) of duplicate and homologous *GATA* gene pairs of different *Ipomoea* species were calculated using TBtools software (Chen et al., 2023).

### 2.7 RNA extraction and qRT-PCR of *GATA* genes in sweet potato

Sweet potato cultivar Ganshu 8 was used as the experimental material. The 6-week-old potato seedlings, approximately 25 cm long, were cut from the field and cultured in 1/2 Hoagland solution for 7 days to keep them alive. The root, stem, leaf, and petiole of Ganshu 8 were measured to analyze the expression specificity of *IbGATA* genes in different tissues. One-week-old seedlings in 1/2 Hoagland solution were treated with 200 mM NaCl and 20% PEG-6000 to evaluate the response of *IbGATA* genes to abiotic stress. The leaves were collected at 0, 6, 12, and 24 h posttreatment, and the untreated plants were used as controls (Zhang et al., 2019). Total RNA from sweet potato leaves was extracted using the FastPure^®^ Plant Total RNA Isolation Kit (Wuhan, China) according to the manufacturer’s instructions. The first-strand cDNA was synthesized using EasyScript^®^ All-in-One First-Strand cDNA Synthesis SuperMix for qPCR (one-step gDNA removal) (Wuhan, China). Each 20 μL contained 4 μL 5× EasyScript^®^ Uni All-in-One SuperMix for qPCR, 1 μL gDNA remover, 1 μg total RNA, and variable RNase-free water. The cycling conditions for PCR were as follows: 42°C for 15 min and then 80°C for 5 s. qRT-PCR was performed using TransStart^®^ Tip Green qPCR SuperMix (Wuhan, China), and each 20 μL mixture contained 10 μL TransStart^®^ Tip Green qPCR SuperMix, 0.8 μL each specific primer, 7.4 μL nuclease-free water, and 1 μL cDNA. The qRT-PCR program comprised preheating at 94°C for 2 min, followed by 45 cycles of denaturation at 94°C for 5 s and annealing at 58°C for 30 s. The expression levels of *IbGATA* genes were detected using qRT-PCR analysis conducted on the LightCycler^®^ 96 system (Roche, United States). Each experiment had three biological replicates and three technical replicates, and the relative expression levels of *GATA* genes were calculated using the 2^−ΔΔCt^ method (Zhang et al., 2019). The sweet potato *β-actin* gene was used as an internal reference gene. The primers used for qRT-PCR in this study are listed in Supplementary Table S1.

### 2.8 Statistical analysis

Data analysis in this study was performed using Microsoft Excel 2019 and SPSS 26 software. Significance of differences between treatments was determined using one-way ANOVA. An LSD test was used to calculate *p*-values, and *p* < 0.01 indicates significant differences.

## 3 Results

### 3.1 Identification of *GATAs* in *Ipomoea* species

A total of 35 *IbGATA* genes were identified in the whole genome of sweet potato, and 33, 34, 39, 63, and 56 *GATA* genes were identified from *Ipomoea aquatica*, *Ipomoea cairica*, *Ipomoea nil*, *Ipomoea triloba*, and *Ipomoea trifida*, respectively (Supplementary Table S2). The amino acid length, MW, theoretical pI, instability index, aliphatic index, and grand average of hydropathicity (GRAVY) of *Ipomoea* species are shown in Supplementary Table S1. These *GATA* genes were named *IbGATA1* to *IbGATA35*, *IaGATA1* to *IaGATA33*, *IcGATA1* to *IcGATA34*, *InGATA1* to *InGATA39*, *ItbGATA1* to *IbGATA63*, and *ItfGATA1* to *ItfGATA56*. In the GATA protein of *Ipomoea batatas*, the length of protein sequences and MW ranged from 142 to 546 aa and 16212.38 to 191512.3 Da, respectively, and the average length and MW were 304 aa and 38161.27 Da, respectively. GRAVY ranged from −1.080 to −0.339, and pI ranged from 5.60 to 10.33. Subcellular localization prediction results showed that all IbGATAs may have nuclear localization signals.

The average lengths of proteins in *I. aquatica*, *I. cairica*, *I. nil, I. triloba*, and *I. trifida* were 328, 351, 291, 304, and 308 aa, respectively. Subcellular localization predictions showed that GATA proteins may have nuclear localization signals, except IcGATA22.

### 3.2 Phylogenetic analysis of *GATA* genes

**FIGURE 1:**
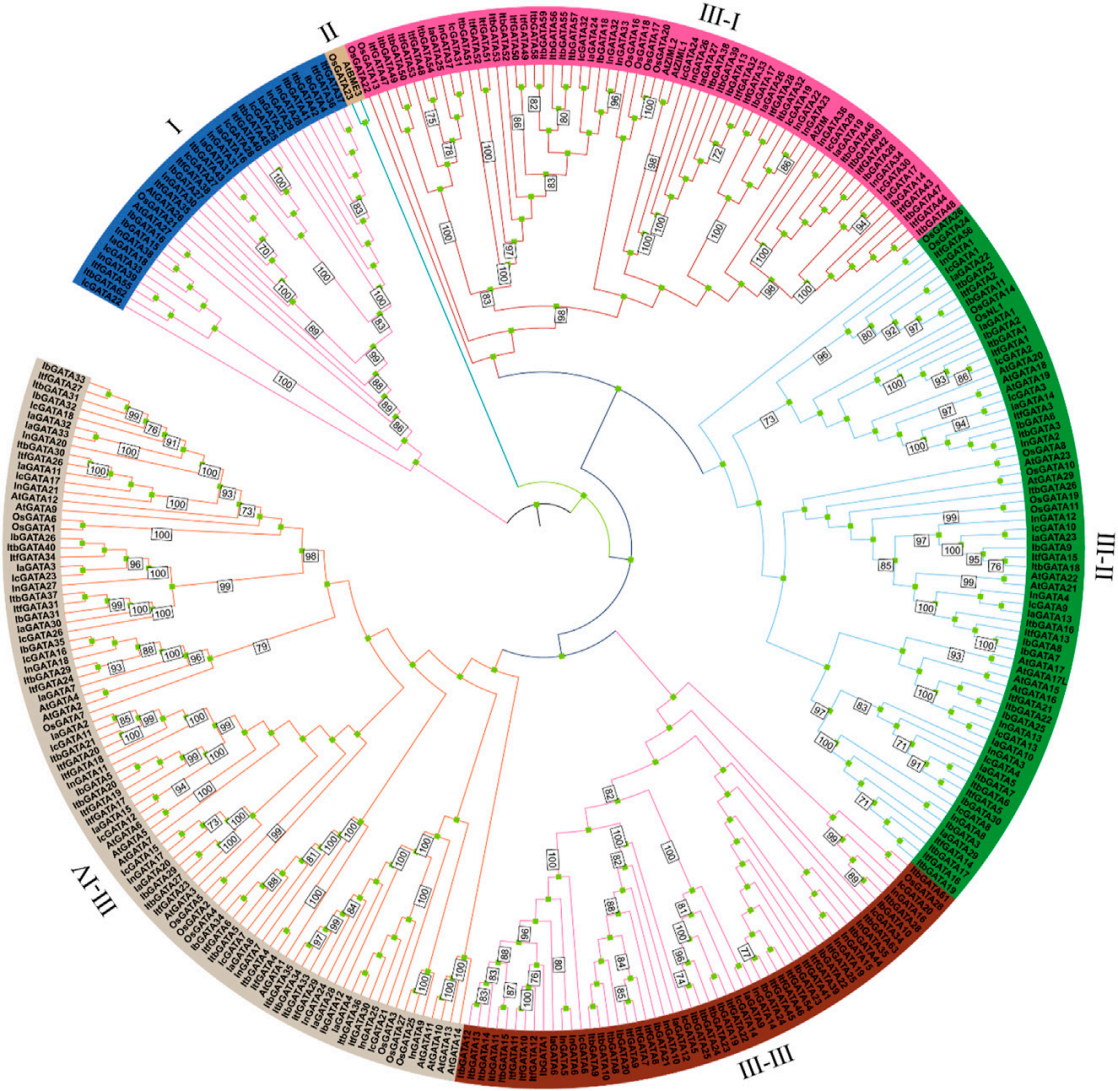
Phylogenetic tree of *GATA* genes in *I. batatas*, *I. aquatica*, *I. cairica*, *I. nil*, *I. triloba*, *I. trifida*, *Arabidopsis thaliana*, and *Oryza sativa*.

To explore the phylogenetic relationship of the *GATA* genes in *Ipomoea* species, a phylogenetic tree was constructed using 316 GATA amino acid sequences from *Arabidopsis thaliana*, *Oryza sativa*, and *Ipomoea* species (Figure 1). It shows that among the different species, the evolutionary tree was clustered into three distinct groups, namely Ⅰ–Ⅲ, with group Ⅲ containing four subclasses: Ⅲ-Ⅰ, Ⅲ-Ⅱ, Ⅲ-Ⅲ, and Ⅲ-Ⅳ. Except for group Ⅱ, all other groups contained *I. batatas*, *I. aquatica*, *I. cairica*, *I. nil*, *I. triloba*, *I. trifida*, *Arabidopsis thaliana*, and *Oryza sativa* GATA proteins, suggesting that the characteristics of the *GATA* gene family emerged prior to the divergence of these species. Among these six groups, groups Ⅲ–Ⅳ exhibited the largest number of GATA proteins, reaching 91, followed by group Ⅲ-Ⅱ (72 GATA proteins), Ⅲ-Ⅰ (65 GATA proteins), Ⅲ-Ⅲ (53 GATA proteins), Ⅰ (33 GATA proteins), and Ⅱ (2 GATA proteins).

### 3.3 Conserved motif and gene structure analysis of GATA proteins

**FIGURE 2:**
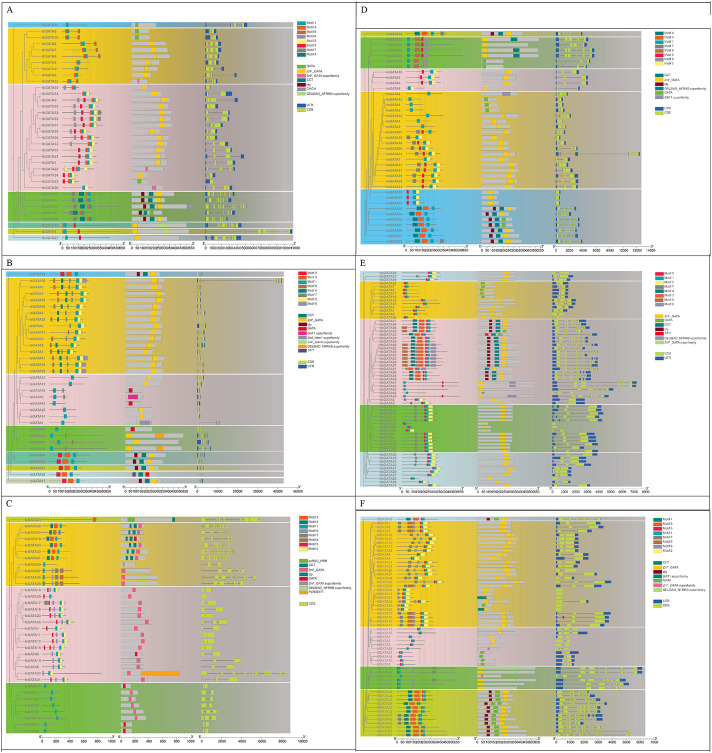
Evolutionary relationship, conserved motifs, protein conserved domains, and gene structure of GATA proteins in *Ipomoea* species. **(A–F)** *I. batatas*, *I. aquatica*, *I. cairica*, *I. nil*, *I. triloba*, and *I. trifida*.

The conserved motifs and gene structure of the GATA family in *Ipomoea* species were analyzed using the MEME online tool to investigate their functional evolution (Figure 2). A total of eight motifs (motif 1 to motif 8) were identified in the six *Ipomoea* species GATA proteins. In *Ipomoea batatas*, motif 1 is the most prevalent. Analysis of the motif distribution in each protein revealed that with the exception of IbGATA15 (containing only one motif), all other IbGATA proteins possess two or more motifs. Furthermore, motif 1 exists in all *IbGATAs*, suggesting that motif 1 constitutes an evolutionarily critical domain in the *IbGATA* genes. In the exon/intron structure, it was found that with the exception of *IbGATA10*, *IbGATA23*, and *IbGATA24*, which contain only one exon, all other genes possess two or more exons and introns. This study further analyzed the distribution of motifs and the structural characterization of *GATA* genes in *I. aquatica*, *I. cairica*, *I. nil*, *I. triloba*, and *I. trifida* (Figures 2B–F). This study aims to establish a foundational framework for elucidating the structural characteristics of *GATA* genes in sweet potato and other *Ipomoea* species.

### 3.4 Chromosomal location and duplication analysis of *GATA* genes

**FIGURE 3:**
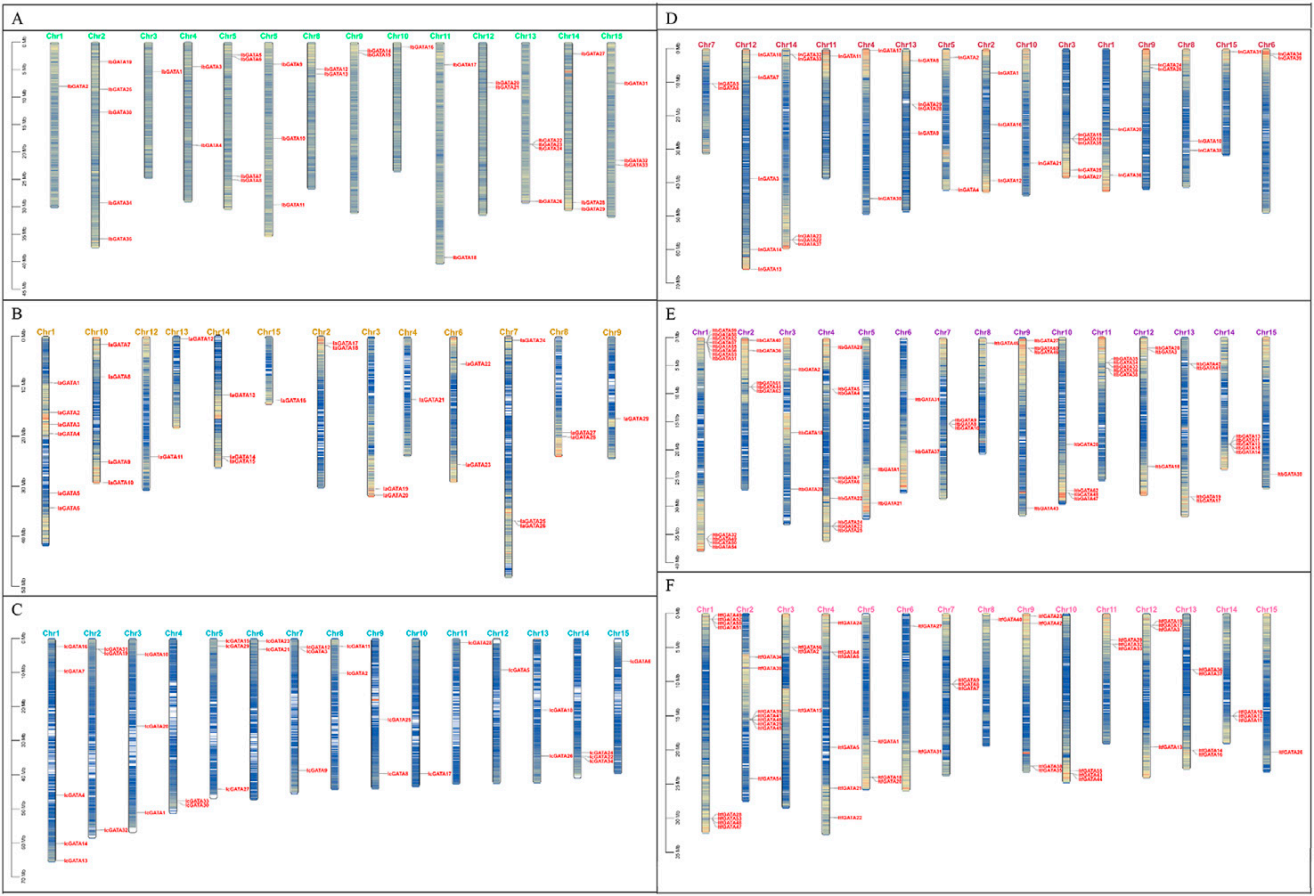
Chromosome localization of *GATA* genes in *Ipomoea* species. **(A–F)** *Ipomoea batatas*, *I. aquatica*, *I. cairica*, *I. nil*, *I. triloba*, and *I. trifida*.

The information about the chromosomal locations of the *GATA* genes was extracted from the *Ipomoea* species genome annotation file, and a chromosomal distribution map of *GATA* genes was generated. A total of 35, 29, 34, 39, 63, and 56 *GATA* genes were mapped throughout the chromosomes of *I. batatas*, *I. aquatica*, *I. cairica*, *I. nil*, *I. triloba*, and *I. trifida* (Figure 3). There were four *GATA* genes that were mapped to unassembled scaffolds in *I. cairica* (Figure 3C)*.* The distribution of *GATA* genes across the chromosomes in *I. batatas* is uneven; there were 1, 5, 1, 2, 4, 3, 2, 2, 1, 2, 2, 4, 3, and 3 *GATA* genes mapped on chromosomes 1, 2, 3, 4, 5, 7, 8, 9, 10, 11, 12, 13, 14, and 15 of *I. batatas*, respectively, whereas no *GATA* genes were mapped on chromosome 5 (Figure 3A). The phenomenon of unbalanced chromosomal distribution of *GATA* genes has also been observed in *I. aquatica*, *I. cairica*, *I. nil*, *I. triloba*, and *I. trifida* (Figures 3B–F).

### 3.5 *Cis*-acting elements in promoter regions of *IbGATA* genes

**FIGURE 4:**
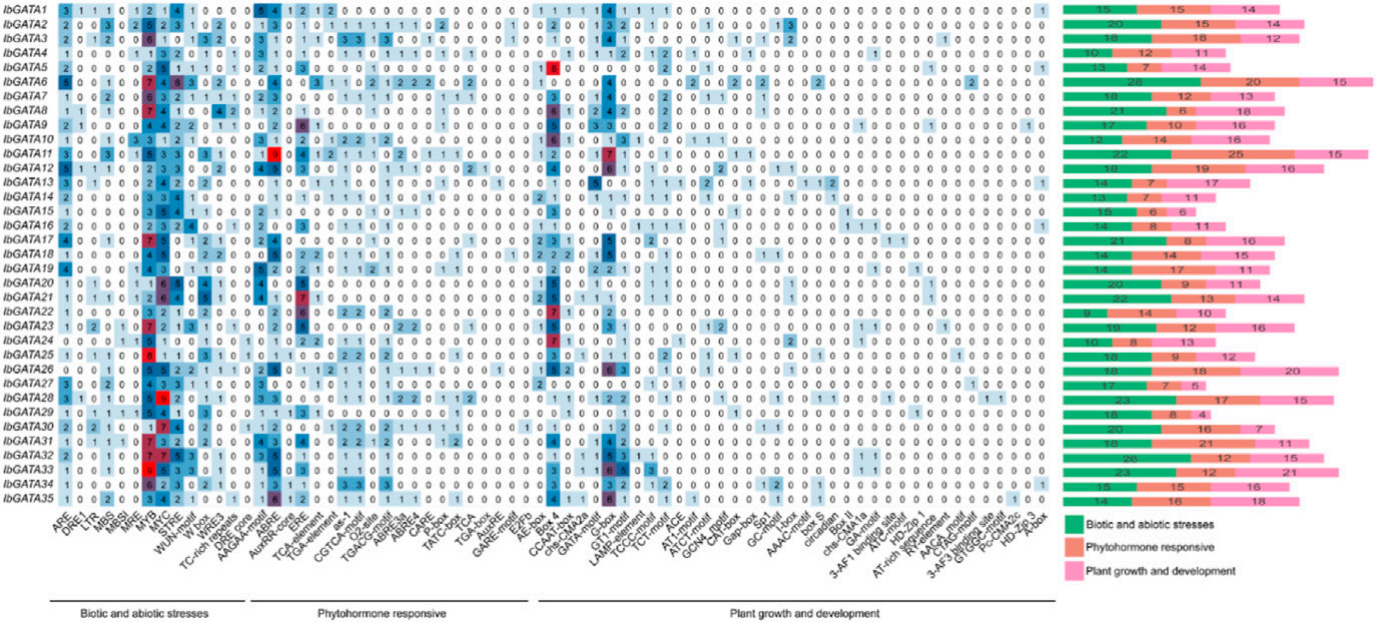
*Cis*-acting elements in the promoters of *IbGATA* genes in *I. batatas.*

To further elucidate the biological functions of the *GATA* gene family in *I*. *batatas*, the promoter sequences of the *IbGATA* genes were analyzed. Various *cis*-acting elements existed in the promoter region of the *IbGATA* genes, such as ABRE, MYB, Box 4, G box, and other elements (Figure 4). The *cis*-acting elements were divided into three types: abiotic and biotic stresses, phytohormone responsive, and plant growth and development. Among the all *cis*-acting elements, MYB has the highest distribution in 35 *IbGATA* genes, which was 153 in total, followed by MYC and Box 4, with 129 and 105, respectively. On the whole, the numbers of biotic and abiotic stresses were significantly greater than those of phytohormone responsive and plant growth and development elements. The results demonstrate that the sweet potato *GATA* gene family may be more sensitive to stress response.

### 3.6 Syntenic analysis of *GATA* genes in *Ipomoea* species

**FIGURE 5:**
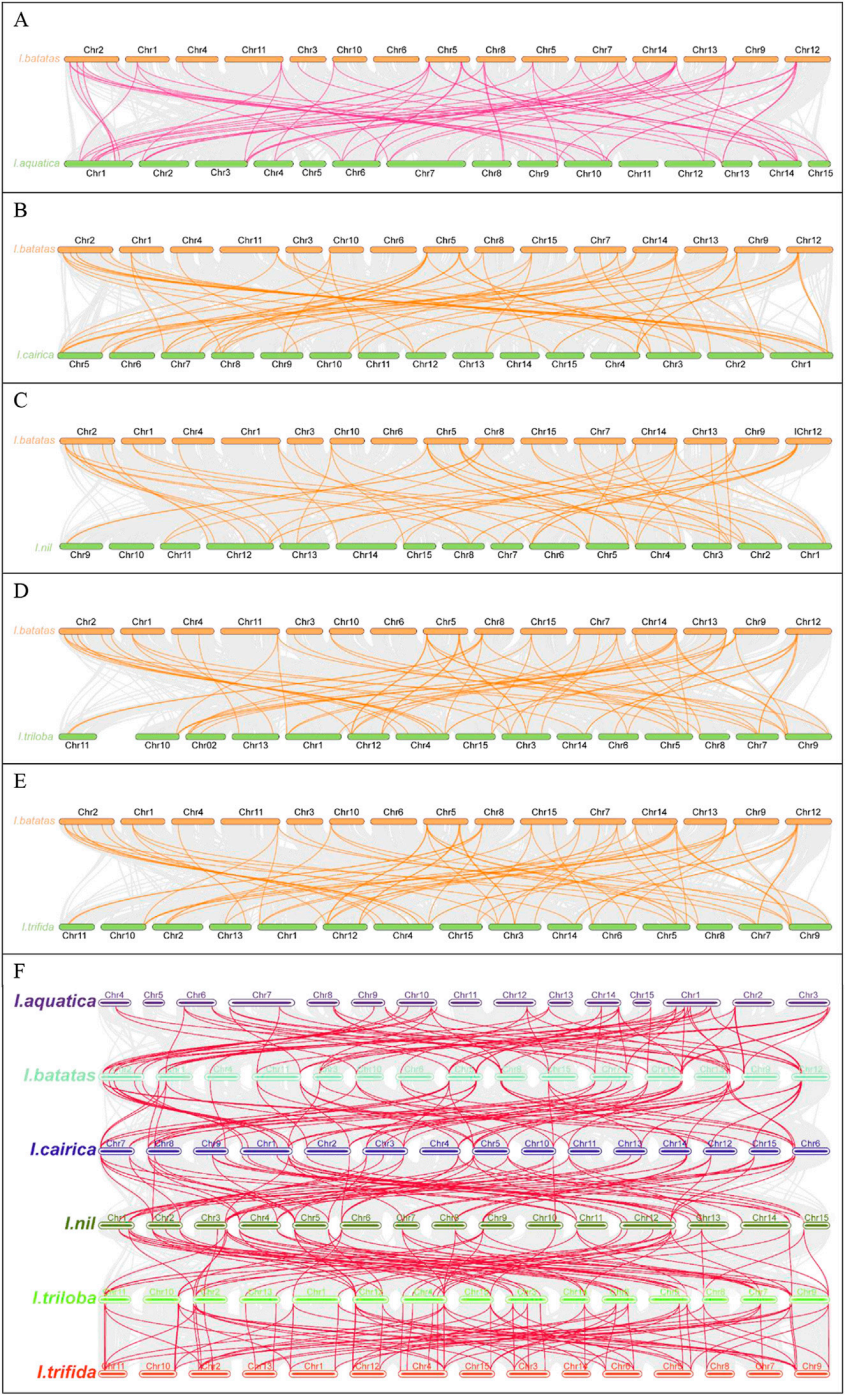
Collinearity analysis of the *GATA* genes between *Ipomoea* species. **(A)** *Ipomoea batatas* and *I. aquatica*; **(B)** *Ipomoea batatas* and *I. cairica*; **(C)** *Ipomoea batatas* and *I. nil*; **(D)** *Ipomoea batatas* and *I. triloba*; **(E)** *Ipomoea batatas* and *I. trifida*. **(F)** Schematic representation of syntenic genes among *I. batatas*, *I. aquatica*, *I. cairica*, *I. nil*, *I. triloba*, and *I. trifida*.

To further systematically elucidate the evolutionary mechanisms of the *IbGATA* family, the collinearity of *IbGATA* gene pairs among the genomes of *I. aquatica*, *I. cairica*, *I. nil, I. triloba*, and *I. trifida* was compared (Figure 5). The results showed that *IbGATA* formed 63, 64, 57, 67, and 67 collinearity gene pairs with *IaGATA*, *IcGATA*, *InGATA*, *ItbGATA*, and *ItfGATA*, respectively.

Multiple *IbGATA* genes have been identified as homologous genes to single *IaGATA*, *IcGATA*, *InGATA*, *ItbGAA*, and *ItfGATA* genes. In addition, there are multiple *IaGATA*, *IcGATA*, *InGATA*, *ItbGAA*, and *ItfGATA* genes that are homogeneous to a single *IbGATA* gene. These results indicate that the GATA gene families of sweet potato and *I. aquatica*, *I. cairica*, *I. nil, I. triloba*, and *I. trifida* share a close evolutionary relationship, and these genes may have similar functions.

**FIGURE 6:**
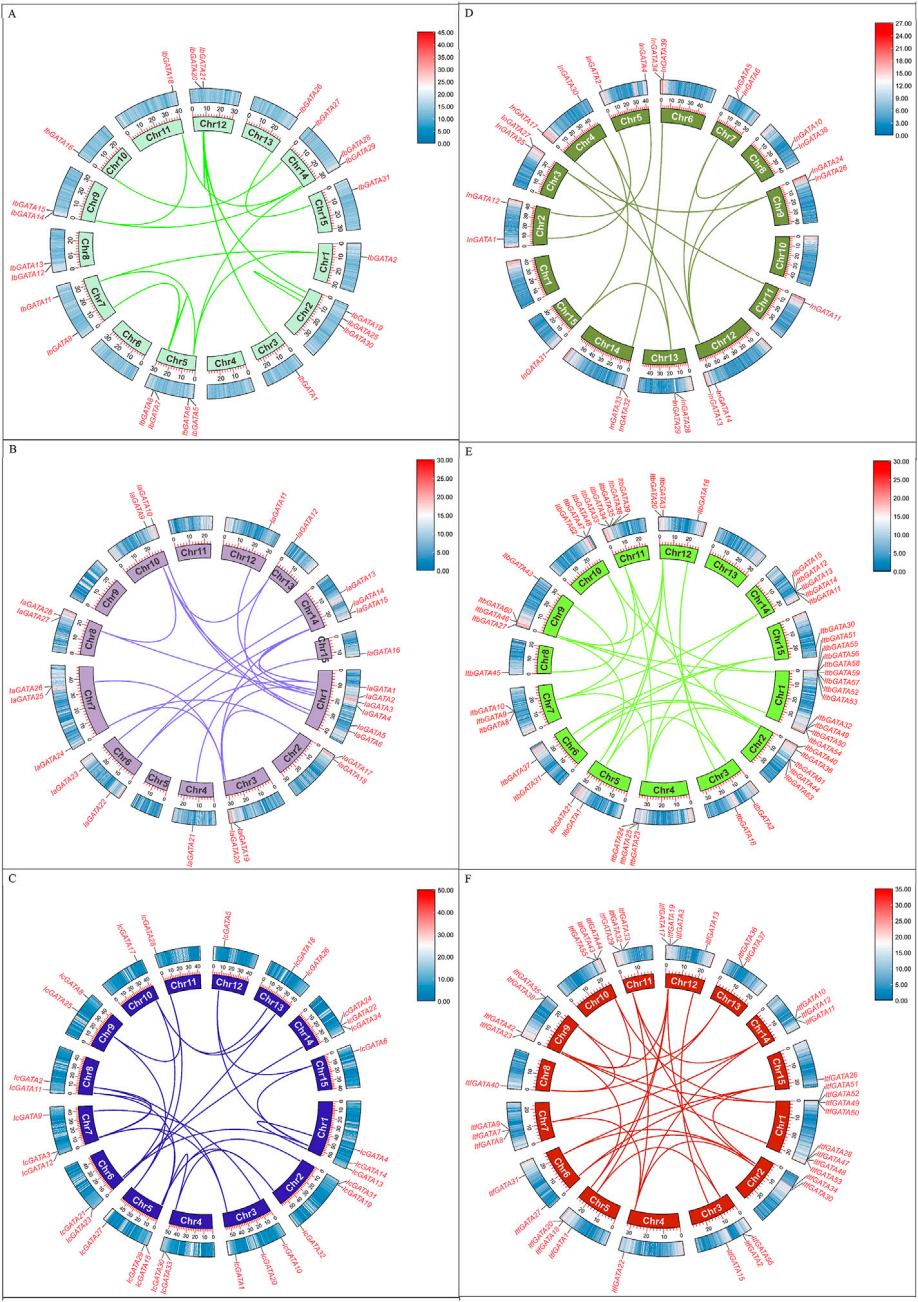
Schematic diagram of *GATA* gene collinearity analysis in *Ipomoea* species. **(A–F)** *I. batatas*, *I. aquatica*, *I. cairica*, *I. nil*, *I. triloba*, and *I. trifida*.

The segmental duplication events of *IbGATA* genes were identified using MCScanX and BLASTp searches (Figure 6). It was found that there were 18 pairs of *IbGATA* genes in the sweet potato chromosome, of which 17 pairs of genes were segmental duplication events in *IbGATA* genes and *IbGATA22/IbGATA23* was a tandem duplication event. The segmental duplication events of *GATA* genes in other *Ipomoea* species were identified, and the results were similar to that of *IbGATA* genes (Figure 6).

### 3.7 Ka/Ks analysis of duplicated and syntenic *GATA* genes

To determine whether the *GATA* genes are under positive selection, the Ka/Ks analysis of syntenic *GATA* genes within six *Ipomoea* species was conducted. In the six *Ipomoea* species, all of the syntenic *GATA* genes possessed a Ka/Ks ratio <1 (Supplementary Table S3). These results suggest that syntenic *GATA* genes were subject to purifying selection in the genome during speciation.

### 3.8 Expression analysis of *IbGATA* genes through qRT-PCR

**FIGURE 7:**
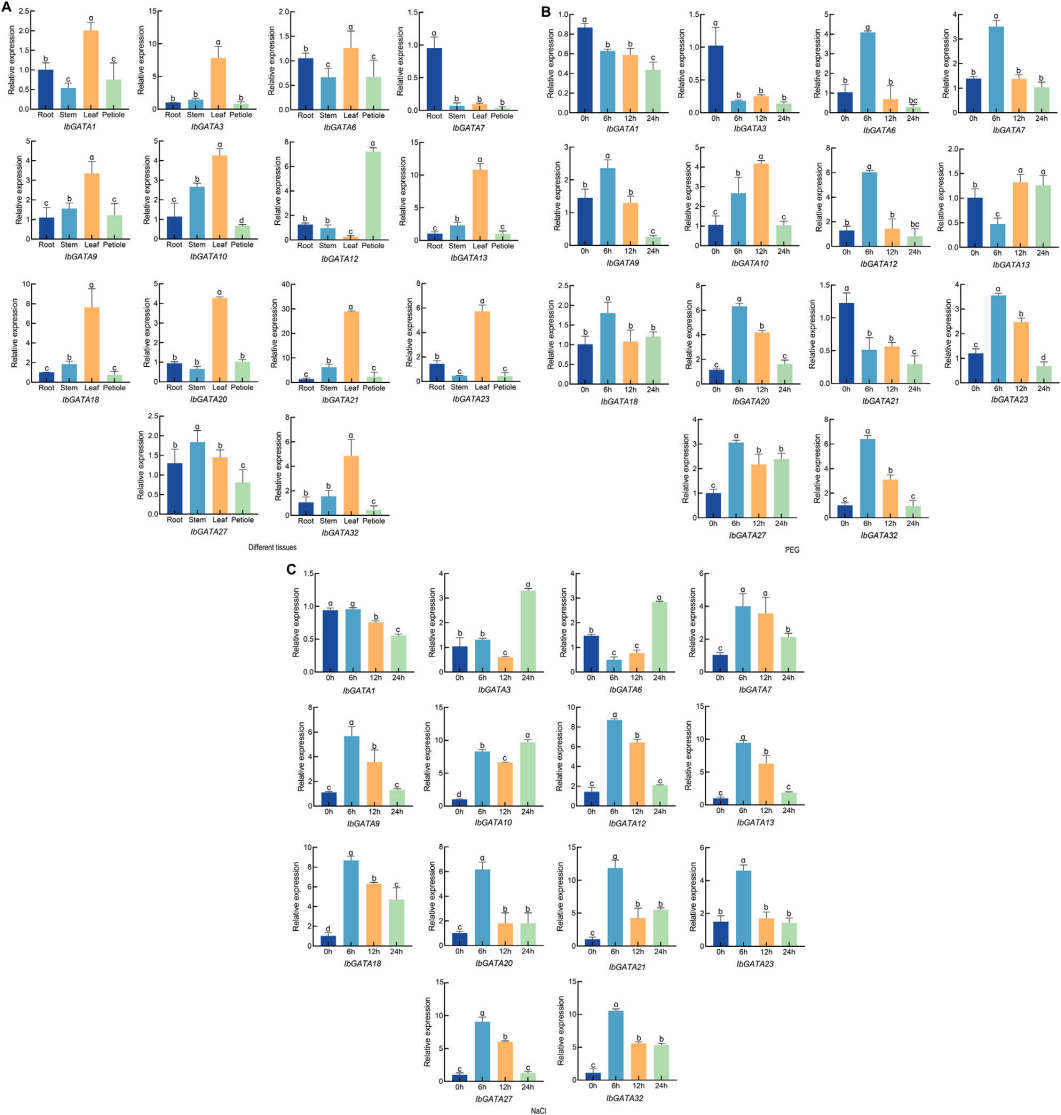
The expression profile of the *IbGATA* gene in sweet potato was detected through qRT-PCR. **(A)** Relative gene expression levels in different tissues: root, stem, leaf, and petiole. **(B)** Relative gene expression levels under drought (20% PEG-6000) treatment over the same time periods (0, 6, 12, and 24 h). The control group was treated with distilled water. **(C)** Relative gene expression levels under salt (200 mM NaCl) treatment over the same time periods (0, 6, 12, and 24 h). Data represent the mean of three biological replicates ±SD (*n* = 3). Error lines indicate standard deviations. Different lowercase letters (a, b, c, and d) on the bars indicate significant differences at *p* < 0.01.

To further investigate the expression characteristics of GATA genes, 17 *IbGATA* genes were chosen in order to study their dynamic expression patterns in different tissues and in response to drought and salt stress (Supplementary Table S2). The expression of *IbGATA* genes in the root, stem, leaf, and petiole was analyzed through qRT-PCR (Figure 7A). The results showed that *IbGATA* expression levels vary across different tissues, and *IbGATA1*, *IbGATA3*, *IbGATA9*, *IbGATA10*, *IbGATA13*, *IbGATA18*, *IbGATA20*, *IbGATA21*, *IbGATA23*, and *IbGATA32* were highly expressed in the leaf of sweet potato. *IbGATA7* showed strong upregulated expression in the root. *IbGATA12* was highly expressed in the petioles. Many *IbGATA* genes, such as *IbGATA13* and *IbGATA21*, had similar expression.

The expression levels of *IbGATA* genes in drought and salt stress were analyzed through qRT-PCR (Figures 7B,C). The expression of *IbGATA6*, *IbGATA7*, *IbGATA9*, *IbGATA10*, *IbGATA12*, *IbGATA18*, *IbGATA20*, *IbGATA23*, *IbGATA27*, and *IbGATA32* was upregulated under drought stress. The expression of *IbGATA6*, *IbGATA7*, *IbGATA9*, *IbGATA12*, *IbGATA18*, *IbGATA20*, *IbGATA23*, *IbGATA27*, and *IbGATA32* was highest at 6 h. In addition, *IbGATA6*, *IbGATA7*, *IbGATA9*, *IbGATA12*, *IbGATA20*, and *IbGATA32* exhibited similar expression patterns under drought conditions. The expression levels of *IbGATA1*, *IbGATA3*, and *IbGATA21* exhibited a downward trend under drought stress (Figure 7B). Under salt stress, the expression of *IbGATA1* was downregulated. By contrast, for example, *IbGATA7* and *IbGATA18* exhibited upregulated expression patterns under salt stress. The expression of *IbGATA3* and *IbGATA6* reached the highest value at 24 h (Figure 7C). These findings indicate that *IbGATA* genes play a significant role in drought and salt stress responses.

## 4 Discussion

GATA TFs have been demonstrated to play important roles in different plant biological processes such as seedling development, signal transduction, nitrogen and carbon metabolism, light regulation, and abiotic stresses (drought, cold, and salinity) (Schwechheimer et al., 2022). The *GATA* gene family has been identified and studied in a variety of plants, including rice (*Oryza sativa*) (Reyes et al., 2004), tomato (*Lycopersicon esculentum*) (Zhao et al., 2021), soybean (*Glycine max*) (Zhang et al., 2020), and potato (*Solanum tuberosum*) (Aksoy et al., 2024). However, primarily due to the fact that widely cultivated sweet potato varieties are highly heterozygous autopolyploid hexaploids with complex genetic analysis challenges and relatively scarce genomic databases, a genome-wide study of the *GATA* gene family has not yet been conducted in sweet potato and other *Ipomoea* species. With the completion of genome sequencing for sweet potato and an increasing number of *Ipomoea* species, these data provide valuable resources for the identification of gene families and genome-wide bioinformatic analyses in sweet potato and other *Ipomoea* species.

In this study, 260 *GATA* genes were identified from sweet potato and other *Ipomoea* species using bioinformatic technology. The number of *GATA* genes were 35, 33, 34, 39, 63, and 56 in sweet potato, *Ipomoea aquatica*, *Ipomoea cairica*, *Ipomoea nil*, *Ipomoea triloba*, and *Ipomoea trifida*, respectively. The expression of these *GATA* genes was similar to that in rice and *Arabidopsis*. The *GATA* gene counts in sweet potato diverges from those of other species, exemplified by *Triticum aestivum* (79) (Zheng et al., 2024), *Dimocarpus longan* Lour (24) (Zheng et al., 2024), and *Setaria italica* (28) (Lai et al., 2022), demonstrating lineage-specific expansion patterns of *GATA* gene families among plant taxa. In addition, the *GATA* genes can be divided into six groups, among which group Ⅲ-Ⅳ has the most members, whereas group Ⅱ has the fewest *GATAs*. The current study provides valuable insights for the future functional characterization of *GATA* genes and contributes to increased adaptive capacity in plants.

In plants, exon/intron structures of *GATA* genes showed a low concentration. In sweet potato, exon numbers in *GATA* genes range from 1 to 8 and exhibit lineage-specific divergence compared to those in *I. aquatica*, *I. cairica*, *I. nil*, *I. triloba*, and *I. trifida.* The exon number in *Ipomoea* is very similar to that of wheat (Feng et al., 2022). The conserved motif analysis revealed that all 35 IbGATA family members contain motif 1, indicating that this motif is crucial for the function of IbGATA proteins. Additionally, different subfamilies contain distinct types of conserved motifs, leading to functional diversification during evolution. In contrast, the conserved motifs of GATA TFs within the same subfamily are generally identical, indicating that these GATA proteins are likely to have similar functions. In brief, IbGATA proteins within the same subfamily share similar conserved motifs, gene structures, and phylogenetic relationships, which enhances the reliability of the subfamily classification of *IbGATA* genes in this study.

*Cis*-acting elements are specific binding sites for TFs, regulating the precise initiation sites and efficiency of gene transcription (Moriwaki et al., 2022). Previous studies have shown that GATA TFs can regulate light signal transduction by binding to elements related to plant growth and development, thereby modulating the light responsiveness within GATA promoter sequences (Luo et al., 2010). It has been found that *CrGATA1* could activate the promoters of light-responsive vindoline pathway genes, and the expression of *CrGATA1* and vindoline pathway genes was greatly induced in *Catharanthus roseus* under light conditions (Liu et al., 2019). In this research, plant growth and development elements, such as light-responsive elements, were widely distributed in *IbGATA* genes, suggesting that *IbGATA* genes could regulate light-response processes in sweet potato. Additionally, the majority of *IbGATA* gene promoters contain hormone-responsive elements, as well as low-temperature and drought stress-responsive elements. The previous studies have found that overexpression of *BdGATA13* in transgenic *Arabidopsis* enhanced drought tolerance compared to the wild type, and *BdGATA13* also promoted primary root development under gibberellins (GAs) treatment (Guo et al., 2021). All 35 *IbGATA* genes contain many *cis*-acting elements related to adverse stress and hormone regulation, which suggests that *IbGATA* genes not only play a vital role in regulating plant growth and development but may also be involved in abiotic stress and hormone regulation.

Gene duplication events are crucial for the expansion and functional diversification of gene families during the evolutionary process (Qiao et al., 2019). In this research, gene duplication events have occurred in *GATA* genes of sweet potato and other *Ipomoea* species during evolution. These findings suggest that segmental duplication events likely represent the predominant mechanism underlying the expansion of the *GATA* gene family during evolution. The collinearity analysis showed that the genomes of sweet potato, *Ipomoea aquatica*, *Ipomoea cairica*, *Ipomoea nil*, *Ipomoea triloba*, and *Ipomoea trifida* have many homologous gene pairs in the *GATA* gene family. The results indicate a closer phylogenetic relationship between the *GATA* gene families of sweet potato and other *Ipomoea* species.

The previous study indicates gene expression patterns can, to some extent, reveal gene function. It has been found that the *DlGATA* genes were strongly upregulated in roots and stems (Zheng et al., 2024). The expression of *TaGATA* genes varies in different tissues of wheat (Feng et al., 2022). In this study, the expression patterns of *IbGATA* genes in different tissues exhibit differential expression. This study revealed a significant variation in the expression levels of *IbGATA* genes across different tissues. For example, *IbGATA1* and *IbGATA9* exhibited markedly higher expression in leaves, *IbGATA7* showed elevated expression in roots, and *IbGATA12* displayed the highest expression in petioles (Figure 7A), suggesting that distinct *IbGATA* genes may function in tissue-specific contexts.

Drought and salt stress are abiotic stress factors that limit the normal growth and development of crops, posing serious threats to land productivity and biomass yield. In tomato, overexpression of *SlGATA17* increases drought tolerance in transgenic plants (Zhao et al., 2021). Overexpression of *TaGATA62* and *TaGATA73* genes significantly enhanced the drought and salt tolerance of yeast and Arabidopsis (Du et al., 2022). In this study, *IbGATA7*, *IbGATA9*, and *IbGATA21* were upregulated under drought and salt stress, suggesting that these genes may function in drought and salt stress signaling pathways contributing to plant drought and salinity tolerance.

## 5 Conclusion

This study systematically analyzed the *GATA* gene family in *Ipomoea* species, including gene structure, predicted physical and chemical properties, conserved domains, collinearity, and evolutionary tree. A phylogenetic tree was constructed using GATA sequences from sweet potato, *Ipomoea aquatica*, *Ipomoea cairica*, *Ipomoea nil*, *Ipomoea trilob*a, *Ipomoea trifida*, rice, and *Arabidopsis*, and the sweet potato *GATA* genes were divided into six groups. In most subfamilies, the exon/intron architecture and motif configurations demonstrated evolutionary conservation. These *GATA* genes were unevenly distributed on 15 chromosomes, and the segmental duplication events were analyzed. The expression characteristics of *GATA* gene family members in various tissues of sweet potato and their stress-responsive expression patterns have been systematically validated through qRT-PCR analysis. This study revealed that GATA TFs play pivotal roles in regulating plant growth and development and mediating stress adaptation mechanisms. In summary, this study systematically deciphered the expression patterns and functional characteristics of the *GATA* gene family in sweet potato and other *Ipomoea* species, offering critical data support for an in-depth understanding of the biological functions of this TF family.

## Data Availability

The datasets presented in this study can be found in online repositories. The names of the repository/repositories and accession number(s) can be found in the article/Supplementary Material.
